# Targeting Indoleamine 2,3-Dioxygenase in Cancer Models Using the Novel Small Molecule Inhibitor NTRC 3883-0

**DOI:** 10.3389/fimmu.2020.609490

**Published:** 2021-01-28

**Authors:** Yvonne Grobben, Jos de Man, Antoon M. van Doornmalen, Michelle Muller, Nicole Willemsen-Seegers, Diep Vu-Pham, Winfried R. Mulder, Martine B. W. Prinsen, Joeri de Wit, Jan Gerard Sterrenburg, Freek van Cauter, Judith E. den Ouden, Anne M. van Altena, Leon F. Massuger, Joost C. M. Uitdehaag, Rogier C. Buijsman, Guido J. R. Zaman

**Affiliations:** ^1^ Netherlands Translational Research Center B.V., Oss, Netherlands; ^2^ Department of Obstetrics and Gynaecology, Radboud University Medical Centre, Nijmegen, Netherlands

**Keywords:** indoleamine 2,3-dioxygenase, cancer immunotherapy, immunosuppression, tryptophan, kynurenine, syngeneic mouse model, ovarian cancer, IDO1 inhibitor

## Abstract

Indoleamine 2,3-dioxygenase (IDO1) is a key regulator of immune suppression by catalyzing the oxidation of L-tryptophan. IDO1 expression has been related to poor prognosis in several cancers and to resistance to checkpoint immunotherapies. We describe the characterization of a novel small molecule IDO1 inhibitor, NTRC 3883-0, in a panel of biochemical and cell-based assays, and various cancer models. NTRC 3883-0 released the inhibitory effect of IDO1 on CD8-positive T cell proliferation in co-cultures of IDO1-overexpressing cells with healthy donor lymphocytes, demonstrating its immune modulatory activity. In a syngeneic mouse model using IDO1-overexpressing B16F10 melanoma cells, NTRC 3883-0 effectively counteracted the IDO1-induced modulation of L-tryptophan and L-kynurenine levels, demonstrating its *in vivo* target modulation. Finally, we studied the expression and activity of IDO1 in primary cell cultures established from the malignant ascites of ovarian cancer patients. In these cultures, IDO1 expression was induced upon stimulation with IFNγ, and its activity could be inhibited by NTRC 3883-0. Based on these results, we propose the use of ascites cell-based functional assays for future patient stratification. Our results are discussed in light of the recent discontinuation of clinical trials of more advanced IDO1 inhibitors and the reconsideration of IDO1 as a valid drug target.

## Introduction

The essential amino acid L-tryptophan (Trp) is an important regulator of cancer progression due to its regulatory role in immune cell activity (1, 2). Depletion of Trp in the tumor microenvironment results in T cell anergy and inhibition of natural killer cell activity (1, 2). The catabolism of Trp is regulated by two distinct, evolutionary unrelated enzymes, indoleamine 2,3-dioxygenase 1 (IDO1) (EC 1.13.11.42), and tryptophan 2,3-dioxygenase (TDO) (EC 1.13.11.11). Both enzymes catalyze the oxidation of Trp, resulting in the formation of N-formyl kynurenine (NFK), which is rapidly converted into kynurenine (Kyn) by the enzyme kynurenine formamidase.

IDO1 and TDO have a distinct tissue distribution and carry out different physiological roles. IDO1 is broadly expressed at low levels in normal tissues (3), but is strongly induced by pro-inflammatory stimuli, such as IFNγ (4). In this manner, the expression of IDO1 by various cell types, including immune, endothelial and epithelial cells, provides a control mechanism to dampen the immune response (5, 6). In contrast, TDO is constitutively expressed at high levels in the liver, where it functions to maintain Trp homeostasis (7). Consistent with this role, TDO has a relatively low affinity for Trp (*K*_M,Trp_, 190 µM) in comparison to IDO1 (*K*_M,Trp_, 6 µM) (8).

In cancer, expression of IDO1 has been observed in both tumor and immune cells. IDO1 expression has been correlated with poor prognosis, increased progression and reduced survival in several cancers (9, 10). Moreover, it has been related to resistance to anti-PD-1 and anti-PD-L1 immunotherapies (10, 11). In the past decade, several small molecule IDO1 inhibitors have been developed to enhance the efficacy of immunotherapy (12). The first-in-class and most advanced IDO1 inhibitor is epacadostat (INCB024360), which is a reversible, substrate-competitive enzyme inhibitor (13). More recently, increasingly potent IDO1 inhibitors have been described which act by displacing the heme cofactor of the enzyme, as exemplified by linrodostat (BMS-986205) (14). Phase I clinical studies with epacadostat have demonstrated that IDO1 inhibition is well tolerated in human patients and results in dose-dependent reductions of Kyn levels and Kyn/Trp ratios in plasma (15). In a phase I combination trial with the anti-PD-1 immunotherapeutic pembrolizumab, objective response was seen in 12 out of 22 melanoma patients treated with epacadostat (16). However, a large phase III combination trial with pembrolizumab (ECHO-301/KEYNOTE-252) was terminated prematurely, since there was no clinical benefit of the combination over pembrolizumab plus placebo (17).

Here we describe NTRC 3883-0, a potent, selective and orally bioavailable IDO1 inhibitor, which is structurally distinct from existing IDO1 inhibitors (12). NTRC 3883-0 was profiled side-by-side with epacadostat in biochemical and cell-based assays for human (h) and mouse (m) IDO1, and the selectivity target TDO. Its immunomodulatory activity was studied *in vitro* in a co-culture assay of an *hIDO1*-overexpressing cell line with lymphocytes. Modulation of IDO1 activity *in vivo* was studied in a syngeneic mouse model of melanoma induced with *mIDO1*-overexpressing B16F10 cells. Finally, we studied the expression of IDO1 and its modulation by NTRC 3883-0 in *ex vivo* primary cell cultures established from the malignant ascites of ovarian cancer patients.

## Materials and Methods

### Inhibitors

The synthesis of NTRC 3883-0 and analogues (**Table 1**) is described in patent application WO2017/153459 (18), where NTRC 3748-0 and NTRC 3883-0 are referred to as Example 27 and 142b, respectively. For preparative purification of NTRC 3883-0, a chirally pure acid (*i.e.*, (*S*)-(+)-2-phenylbutyric acid) was introduced at the hydroxyl amide function of NTRC 3748-0, followed by separation of the two diastereoisomers by straight phase column chromatography. Deprotection of the chiral auxiliary resulted in **3k** and NTRC 3883-0 (**Table 1**). The absolute configuration of the stereogenic center in NTRC 3883-0 was established by single-crystal X-ray diffraction, which additionally confirmed the orientation of the *o*,*o*-difluorobenzoyl group (**Figure S1**). Epacadostat was purchased at PharmaBlock (#PBLJ9203). The selective TDO inhibitor NTRC 3531-0 was synthesized as described in patent application WO2018/011227 A1 (19).

**Table 1:** Structure-activity relationship of NTRC 3883-0 and analogues.

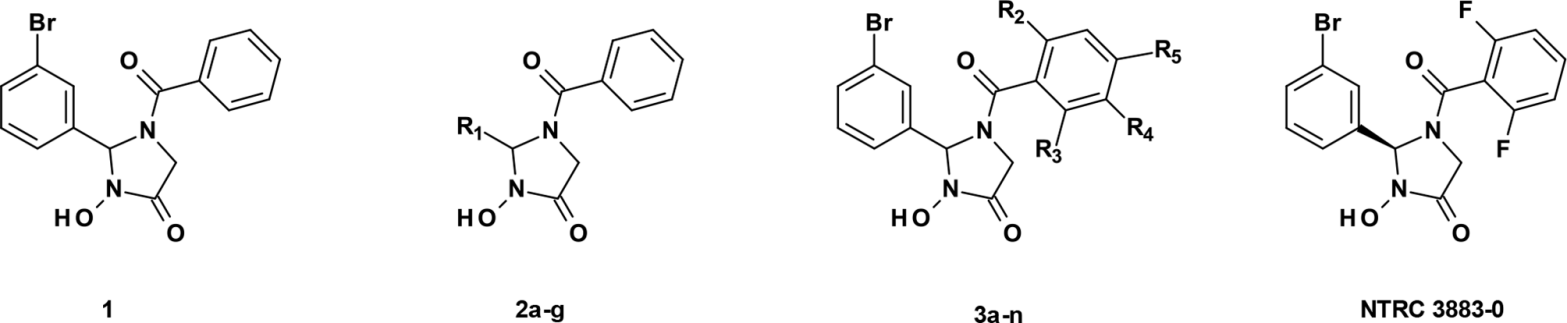								
Compound	Configuration	R_1_	R_2_	R_3_	R_4_	R_5_	IC_50_ IDO1 (nM)	IC_50_ A375 (nM)
1	*R/S*	3-Br-Ph	–	–	–	–	1,220	4,520
2a	*R/S*	Ph	–	–	–	–	> 31,600	> 31,600
2b	*R/S*	3-Cl-Ph	–	–	–	–	2,380	5,220
2c	*R/S*	2-Br-Ph	–	–	–	–	> 31,600	> 31,600
2d	*R/S*	4-Br-Ph	–	–	–	–	> 31,600	> 31,600
2e	*R/S*	cyclohexyl	–	–	–	–	> 31,600	> 31,600
2f	*R/S*	3-CN-Ph	–	–	–	–	17,900	> 31,600
2g	*R/S*	3-CF_3_-Ph	–	–	–	–	2,460	5,290
3a	*R/S*	–	H	H	H	F	2,170	11,000
3b	*R/S*	–	H	H	F	H	910	5,240
3c	*R/S*	–	F	H	H	H	479	1,150
3d	*R/S*	–	CH_3_	H	H	H	4,020	21,700
3e	*R/S*	–	OCH_3_	H	H	H	> 31,600	> 31,600
3f	*R/S*	–	Cl	H	H	H	784	4600
3g	*R/S*	–	Br	H	H	H	1,780	12,500
3h	*R/S*	–	F	H	F	H	600	2,830
3i	*R/S*	–	F	H	H	F	582	2,140
3j (NTRC 3748-0)	*R/S*	–	F	F	H	H	198	589
3k	*R*	–	F	F	H	H	2,170	4,590
NTRC 3883-0	*S*	–	F	F	H	H	123	182

NTRC 3883-0 and its analogues were developed by medicinal chemistry optimization from a 3-hydroxyimidazolin-4-one hit compound (1) identified by ultra-high throughput screening. Inhibitory potencies (IC_50_) were determined in a human IDO1 biochemical assay and a cell-based assay with IFNγ-stimulated A375 human melanoma cells. All analogues were inactive in a biochemical assay for TDO (IC_50_ > 31.6 µM).

### IDO1, TDO, and CYP Biochemical Assays

Full-length recombinant human and mouse IDO1 were expressed in *Escherichia coli* with a hexahistidine tag at the N-terminus, and purified by affinity chromatography to > 95% purity, as described (8). Human and mouse TDO containing a C-terminal hexahistidine tag were analogously expressed and purified to > 95% purity (8). The inhibitory activity of compounds was determined with the NFK GreenScreen assay technology under the conditions as described (8), except for the reaction buffer for IDO1, which was replaced by 50 mM NaH_2_PO_4_, pH 7.0, supplemented with 0.01% Tween-20 and 1% glycerol. Inhibition of the CYP3A4 and CYP2D6 enzymes was determined with the P450-Glo™ Screening System with Luciferin-IPA (Promega, #V9920 and V9890). For determination of IC_50_ values, the effect of inhibitors was determined in 10-point duplicate dose-response curves. Graphs were fitted to a four-parameter logistics equation in XLfit (ID Business Solutions, Ltd., Guildford, UK) from which IC_50_ values were calculated.

### Cancer Cell Lines

The A375 and SW48 cancer cell lines were purchased from the American Type Culture Collection (ATCC, #CRL-1619, RRID: CVCL_0132 and #CCL-231, RRID: CVCL_1724) (Manassas, VA, USA) and cultured in the ATCC-recommended media. All experiments with non-recombinant cell lines were carried out within ten passages from the original vials of ATCC who authenticated the cell lines by short tandem repeat analysis.

### Recombinant Cell Lines

The GripTite™ 293 MSR cell line was purchased from ThermoFisher (#R79507, RRID: CVCL_U428), the B16F10 cell line from ATCC (#CRL-6475, RRID: CVCL_0159), and the GL-261 cell line from the Deutsche Sammlung von Mikroorganismen und Zellkulturen (DSMZ, #ACC 802, RRID: CVCL_Y003). Each cell line was cultured in the medium recommended by the supplier, with FBS replaced by BCS for the B16F10 and GL-261 cell lines. Sublines of GripTite™ 293 MSR stably overexpressing human *IDO1* (HEK-hIDO1), GripTite™ 293 MSR stably overexpressing human *TDO2* (HEK-hTDO), B16F10 stably overexpressing mouse *IDO1* (B16F10-mIDO1), and GL-261 stably overexpressing mouse *TDO2* (GL-261-mTDO) were generated by transfection of the respective cells with respectively full-length *hIDO1*, *hTDO2*, *mIDO1*, or *mTDO2* cDNA cloned in the expression vector pEF6v5 (Thermo Fisher, #V96120). Cells were transfected with Lipofectamine 3000 (Thermo Fisher) and single cell clones were selected by blasticidin selection and limited dilution in 384-well culture plates. Trp-catabolizing activity was evaluated using NFK Green or 4-(dimethylamino)benzaldehyde (pDMAB) (20). To assess the stability of *IDO1* or *TDO2* expression, clones were cultured for 4 weeks in the absence of blasticidin, during which they were tested weekly for Trp-catabolizing activity. Afterwards, the mRNA and protein levels, and the inhibitory potency of reference compounds were determined in the resulting sublines. Cell-based assays with the transfected cell lines were performed in the absence of blasticidin.

### Cell-Based Assays

Inhibition of the Trp-catabolizing activity in A375 cells, HEK-hTDO cells, GL-261-mTDO cells and primary patient samples (all seeded at 8000 cells/well) was determined after incubation of the cells with compound for 1 h, followed by supplementation of the culture medium with 200 µM Trp and incubation for 42 h. The A375 cells and primary patient samples were stimulated with 200 ng/ml IFNγ prior to the incubation period. The remaining cell-based assays were performed analogously to the HEK-hTDO and GL-261-mTDO assays, with the following exceptions. For the SW48 cells, incubation with compound and Trp was performed for 18 h. For the HEK-hIDO1 cells, the cells were seeded at 16,000 cells/well, and no additional Trp was added to the culture medium. For the B16F10-mIDO1 cells, no additional Trp was added to the culture medium, and the cells were incubated for 66 h with compound. Inhibition of the Trp-catabolizing activity in heparinized human whole blood was determined after three-fold dilution of the blood in RPMI 1640 supplemented with 1% penicillin/streptomycin (P/S), incubation of the diluted blood with compound for 1 h, addition of 800 µM Trp and 200 ng/ml IFNγ, and incubation for 66 h.

For the A375, SW48, HEK-hIDO1, and HEK-hTDO cell-based assays, the Trp-to-NFK conversion was measured with NFK Green, as described previously (8). For the B16F10-mIDO1 cells, GL-261-mTDO cells, and heparinized human whole blood, the Trp-to-NFK conversion was measured with pDMAB (20). Briefly, 5% trichloroacetic acid in Milli-Q water was added to each well, followed by incubation at 55°C for 1 h, centrifugation for 20 min at 2,900 x g, transfer of the supernatant to a new plate, and addition of 2% pDMAB (Fisher Chemicals) dissolved in acetic acid to each supernatant. After incubation for 10 min at room temperature, the formation of L-kynurenine was determined by measurement of the absorbance at 480 nm. In parallel with the cell-based NFK Green assays, the cytotoxicity of the compounds was determined in cell viability assays using ATPlite™ 1Step (PerkinElmer) (8). NTRC 3883-0, epacadostat and NTRC 3531-0 were not cytotoxic to any cell line used in this study.

### Gene Expression Analysis

RNA was isolated from cell cultures and tumors with the RNeasy Mini kit (Qiagen). cDNA was prepared using the QuantiTect Reverse Transcription kit (Qiagen) and quantitative real-time PCR (qPCR) was performed in a Bio-Rad CFX96 cycler using SYBR™ Select Master Mix (Thermo Fisher) following standard protocols (21). The sequences of the qPCR primers are provided in **Table S1**.

### Immunoblot Analysis

The expression of hIDO1, mIDO1, and hTDO in cell cultures was determined by SDS-PAGE and immunoblot analysis. hIDO1 and mIDO1 were detected with anti-IDO1 rabbit monoclonal antibody (Cell Signaling, #86630, RRID: AB_2636818), HRP-conjugated anti-rabbit IgG (Cell Signaling, #7074, RRID: AB_2099233) and enhanced chemiluminescence (ECL) using Clarity™ Western ECL substrate (BioRad, #170-5060). hTDO was detected with anti-TDO mouse monoclonal antibody (OriGene, #TA504879, RRID: AB_2622669), HRP-conjugated anti-mouse IgG (Cell Signaling, #7076S, RRID: AB_330924), and ECL. To control for equal loading of the samples on SDS-PAGE gels, the blots were stripped using Restore™ Plus Western Blot Stripping Buffer (ThermoFisher, #46430), after which β-actin was detected using anti-β-actin rabbit polyclonal antibody (Cell Signaling, #4967, RRID: AB_330288), HRP-conjugated anti-rabbit IgG (Cell Signaling) and ECL.

### Co-Culture Assay

HEK-hIDO1 cells were seeded at high density (25,000 cells per well) in clear 96-well culture plates (Greiner, #651101) in RPMI 1640 medium without Trp (PAN Biotech, Germany, #P04-17598), supplemented with 10% FBS and 1% P/S. Trp was added to a final concentration of 7.5 µM, or 200 µM as a control. NTRC 3883-0 or epacadostat was added to a final concentration of 10 or 1 µM, respectively. The next day, PBMCs isolated from a buffy coat were labeled with 0.5 µM CFSE in PBS at 37°C in the dark for 13 min. The labeling reaction was stopped with complete RPMI 1640 medium, supplemented with 10% FBS and 1% P/S. After washing twice with medium, the PBMCs were stimulated with αCD2/αCD3/αCD28-coupled microbeads (Miltenyi, #130-091-441) at a bead-to-cell ratio of 1:1, and added to the HEK-293 cells at a density of 100,000 cells per well. After co-culturing for 5 days, the PBMCs were transferred to a V-bottom plate (Greiner, #651101), washed twice with ice-cold PBS containing 0.5% bovine serum albumin (BSA) (Sigma-Aldrich, #A7906-100G) and incubated for 10 min at 4°C with Fc-receptor blocking reagent (Miltenyi, #130-059-901). Without washing the PBMCs, αCD8-PE (Miltenyi, clone REA734, #130-110-678, RRID: AB_2659235) and REA-S isotype control (Miltenyi, #130-113-439, RRID: AB_2733012) were added. After incubation for 10 min at 4°C, the PBMCs were washed twice with 0.5% BSA in PBS and analyzed on a Guave^®^ easyCyte 5HT Benchtop Flow Cytometer (Merck Millipore) with a blue 488 nm laser. Flow cytometry data were analyzed using Kaluza Analysis (Beckman Coulter Life Sciences, version 2.1). Quantitative analysis of the cell proliferation data was performed using flowFit in RStudio (22).

### *In Vivo* Pharmacokinetics and Pharmacodynamics Models

*In vivo* pharmacokinetics studies were performed at Charles River Laboratories (‘s-Hertogenbosch, The Netherlands). *In vivo* pharmacodynamic and intervention studies were performed at ProQinase (Freiburg, Germany) and Charles River Laboratories (Morrisville, NC). All experimental protocols were approved by, and performed in accordance with the guidelines of the Ethics Committee for Animal Experimentation of the respective organizations.

Pharmacokinetic properties of NTRC 3883-0 were determined in plasma following single oral (p.o.) and i.v. administration to male CD-1 mice (n = 6 per treatment group with three mice sampled per time point), Wistar rats (n = 3), cynomolgus monkey (n = 1) and Beagle dogs (n = 3). Formulations were prepared in DMSO, Kolliphor^®^ EL, 5% D-mannitol in Elix water at a volume ratio of 1:1:8 for i.v. administration, and in 0.5% gelatin, 5% D-mannitol in Elix water for p.o. administration. Plasma samples were collected at eight to eleven time points up to 24 h after administration. Pharmacokinetic properties and required dosing in humans were predicted by allometric scaling by Karin Jorga Life Science Consulting (Basel, Switzerland) (**Figure S2** and **Table S2**).

For the first CT26 mouse model study, performed at ProQinase, 5 × 10^5^ CT26 cells were implanted subcutaneously into female BALB/c mice on day 0. From day 8 onwards, 50 mg/kg of NTRC 3748-0 (**Table 1**) or 100 mg/kg of epacadostat was administered p.o., respectively twice daily (b.i.d.) and once daily (q.d.), as a suspension in 0.5% gelatin, 5% mannitol in water, with n = 8 mice per treatment group. Tumor growth was monitored using caliper measurement 2 times weekly. The study was terminated at day 22 based on the tumor burden. Tumors and plasma were sampled 2 h post last dose.

For the second study, performed at Charles River Laboratories, 3 × 10^5^ CT26 cells were implanted subcutaneously into female BALB/c mice on day 0. From day 8 onwards, 100 mg/kg of NTRC 3883-0 or epacadostat was administered p.o. b.i.d. as a suspension in 0.5% gelatin, 5% mannitol in water, with n = 10 mice per treatment group. Tumor growth was monitored using caliper measurement 3 times weekly. A tumor volume of 2000 mm^3^ was used as the endpoint of the experiment for the individual animals. Tumors were sampled 2 h post last dose.

For development of the *mIDO1*-overexpressing B16F10 mouse model, performed at Charles River Laboratories, female B6D2F1 mice were implanted subcutaneously with 1 × 10^6^ parental B16F10 cells or B16F10-mIDO1 cells (subline i6 or j19) on day 0 with n = 10 mice per group. A tumor volume of 2000 mm^3^ was used as the endpoint of the experiment for the individual animals. To determine the optimal inoculation cell concentration, mice were implanted with 3 × 10^5^, 1 × 10^5^ or 3 × 10^4^ parental or *mIDO1*-overexpressing B16F10 cells (subline j19) with n = 5 mice per group. The same endpoint of the experiment was used as described above. This yielded on average no differences in growth rate between the parental and *mIDO1*-overexpressing groups, but instead resulted in a strong reduction of the take rate at the lower inoculation concentrations. For the intervention study, 3 x 10^5^ cells of the B16F10-mIDO1 subline j19 were implanted on day 0, and treatment was started on day 3 with vehicle (DMSO, Kolliphor, 5% mannitol; 10/10/80, v/v/v), 100 mg/kg NTRC 3883-0 or epacadostat administered p.o. b.i.d. with n = 12 mice per treatment group. Four animals were excluded from analysis due to non-treatment-related deaths. Tumors and plasma were sampled 2 h post last dose.

### Quantification of Inhibitor, Trp, and Kyn Levels

Tissues were homogenized in PBS at a ratio of 1 mg per 10 μl using micro pellet pestles mounted on a Micro-Tube Homogenizer System (Thomas Scientific). After centrifugation, proteins were removed from the homogenized tissue supernatants and plasma samples by precipitation with methanol. The concentrations of inhibitor, Trp and Kyn were determined by LC-MS/MS.

### Patient Samples

Ascites was collected from patients with a primary diagnosis of advanced epithelial ovarian cancer during paracentesis for diagnostics or symptom relieve, or during primary debulking surgery. Ascites was filtered and cells were collected by centrifugation as described (23). To enrich for tumor cells, ascites cell samples were allowed to adhere overnight to tissue culture plastic. The cell samples were then cultured for 2–3 weeks in advanced RPMI medium (ThermoFisher), supplemented with 10% FBS, 1% glutamax and 1% P/S, with 2 or 3 times passaging of the cells. qPCR analyses of the *MUC16, HE4, IDO1, and TDO2* genes were performed for all samples as described above. Five samples which could be cultured over multiple passages were analyzed for cell surface expression of mucin-16 and EpCAM by flow cytometry using FITC-conjugated anti-mucin-16 (AssayPro, #32189-05141) and APC-conjugated anti-EpCAM (Miltenyi, #130-111-117, RRID: AB_2657496) to assure that the cell cultures consisted of malignant cells. IDO1 mRNA and protein levels in these samples were analyzed as described above after incubation with or without 200 ng/ml IFNγ for 24 h. The collection of ascites and the research described was conducted with approval of the medical ethical committee of the Radboud university medical center and informed written consent from each subject.

### Statistical Analyses

Differences in plasma Trp and Kyn levels between the B16F10 mouse model groups with and without tumor development during the intervention study were analyzed using the Mann-Whitney U test. Differences in intratumoral Trp and Kyn levels between the intervention groups were analyzed by Welch’s ANOVA followed by Games-Howell *post hoc* analysis. Statistical analyses were performed in RStudio.

## Results

### Discovery of a Novel Class of IDO1 Inhibitors

A 3-hydroxyimidazolin-4-one class of IDO1 inhibitors was identified in a biochemical screen of IDO1 performed by the European Lead Factory. The initial hit, compound 1 in **Table 1**, inhibited IDO1 with a half-maximal inhibitory concentration (IC_50_) of 1.2 µM and was inactive on human TDO at the highest tested concentration of 31.6 µM. Cellular activity was determined by measuring inhibition of Trp-catabolizing activity in the human A375 melanoma cell line, which expresses IDO1 after stimulation with IFNγ. Analogues of compound **1** were synthesized with modifications at five positions (**Table 1**) and isolated as racemic mixtures except for **3k** and NTRC 3883-0. Optimization was started with the synthesis of analogs **2a–g**, in which the 3-bromophenyl moiety was varied. These analogs showed that this position of the molecule possesses a very tight structure-activity relationship, since deletion (**2a**), substitution (**2b**, **f**, and **g**) or an alternative position (**2c** and **d**) of the 3-bromo group resulted in a significant loss of potency. Having established the importance of the 3-bromophenyl pharmacophore, efforts were focused on analogs of the benzoyl moiety (**3a–j** in **Table 1**). Contrary to the introduction of a fluoro group at the *para* (**3a**) or *meta* (**3b**) position, introduction of an *o*-fluoro (**3c**) group resulted in a potency improvement in both the enzymatic and cellular IDO1 assay (**Table 1**). Other substituents at the *ortho* position (**3d–f**) were not tolerated, except for the *o*-chloro substitution, which showed a similar potency, albeit at the expense of a higher molecular weight. Addition of a second fluoro group to **3c** resulted in compounds **3h-j**. The strongest increase in potency was observed for compound **3j**. The additional *o*-fluoro group in **3j** presumably helps to orientate the phenyl group in a favorable position perpendicular to the 3-hydroxyimidazolin-4-one core, thereby reducing the entropy loss upon binding to IDO1. After chiral separation of the two enantiomers of **3j**, NTRC 3883-0 was identified as the more active enantiomer (eutomer).

### *In Vitro* Characterization of NTRC 3883-0

**Figure 1:**
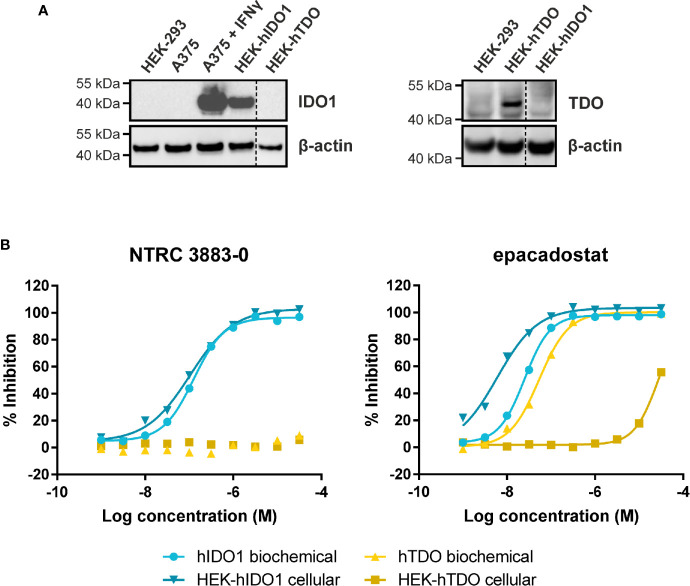
Side-by-side comparison of biochemical and cellular activity of NTRC 3883-0 and epacadostat. **(A)** Immunoblot analysis of the HEK-hIDO1, HEK-hTDO, and IFNγ-stimulated A375 cell lines used for cellular assays. **(B)** Inhibition profile of NTRC 3883-0 and epacadostat in the hIDO1 and hTDO biochemical and HEK-293 cell-based assays.

NTRC 3883-0 inhibited human IDO1 with an IC_50_ of 123 nM and was inactive on TDO (**Table 2**). Trp-catabolizing activity in IFNγ-stimulated A375 cells was inhibited with an IC_50_ of 182 nM, whereas the compound had no effect on Trp-catabolizing activity in the human colorectal carcinoma SW48 cell line, which constitutively expresses TDO (8). Cellular activity was additionally determined in HEK-293 cells stably overexpressing the full-length human *IDO1* (HEK-hIDO1) or *TDO2* cDNA (the gene encoding the TDO enzyme; HEK-hTDO) (**Figure 1A**), since these cells have no detectable endogenous expression of either IDO1 or TDO as determined by qPCR and immunoblot analysis (**Figure 1A**). NTRC 3883-0 inhibited Trp-catabolizing activity in the HEK-hIDO1 cell line with an IC_50_ of 119 nM and had no effect on Trp-catabolizing activity in the HEK-hTDO cells (**Figure 1B**; **Table 2**). The effect on IDO1 activity in normal cells was determined in assays with human whole blood (hWB) from healthy donors after stimulation with IFNγ. In the hWB assay, NTRC 3883-0 inhibited Trp-catabolizing activity with an IC_50_ of 378 nM (**Table 2**).

**Table 2:** *In vitro* characteristics of NTRC 3883-0 and epacadostat.

Assay	NTRC 3883-0	epacadostat
hIDO1	123 (116–130) (n = 85)	27 (25–29) (n = 91)
hTDO	< 20% inhibition @ 31,600 (n = 82)	54 (50–58) (n = 89)
HEK-hIDO1	119 (n = 2)	7.9 (n = 2)
HEK-hTDO	< 20% inhibition @ 31,600 (n = 2)	24,100 (n = 2)
A375 + IFNγ (hIDO1)	182 (155–214) (n = 63)	20 (18–22) (n = 70)
SW48 (hTDO)	< 20% inhibition @ 31,600 (n = 19)	6,460 (5,650–7,390) (n = 24)
hWB + IFNγ (hIDO1)	378 (349–408) (n = 12)	54 (48–62) (n = 12)
mIDO1	93 (n = 2)	39 (30–49) (n = 4)
mTDO	< 20% inhibition @ 31,600 (n = 2)	225 (102–492) (n = 3)
B16F10-mIDO1	18 (n = 2)	88 (n = 2)
GL-261-mTDO	< 20% inhibition @ 31,600 (n = 2)	28% inhibition @ 31,600 (n = 2)

Potency (IC_50_) in nM of IDO1 inhibitors NTRC 3883-0 and epacadostat in biochemical and cell-based assays for human (h) and mouse (m) IDO1 or TDO. 95% confidence intervals and number of experimental replicates (n) are given within brackets. hWB, human whole blood.

The clinical IDO1 inhibitor epacadostat (13) was profiled in the same assays for reference purposes (**Table 2**). Our data confirm that epacadostat is a potent IDO1 inhibitor (**Figure 1B**; **Table 2**). However, a striking discrepancy in its selectivity over TDO in biochemical and cell-based assays was noted. Epacadostat potently inhibited TDO in the biochemical assay with an IC_50_ of 54 nM, which is in the same range as its potency in the biochemical assay for IDO1 (**Figure 1B**; **Table 2**). In contrast, epacadostat showed more than 400 times lower activity on TDO in the HEK-hTDO assay, demonstrating that it is selective cellularly.

*In vitro* profiling of NTRC 3883-0 against a panel of 44 pharmacologically relevant receptors, ion channels, transporters and enzymes at a concentration of 10 µM at Eurofins-CEREP (Le Bois L’Evêque, France) revealed no cross-reactivities, further demonstrating the selectivity of NTRC 3883-0. NTRC 3883-0 also did not inhibit the activity of the cytochrome P450 (CYP) enzymes CYP2D6 and CYP3A4 at 10 µM. Since CYPs, like IDO1, contain a heme center, these data demonstrate that NTRC 3883-0 interacts selectively with the active site of IDO1.

### Modulation of Cytotoxic T Cell Proliferation

**Figure 2:**
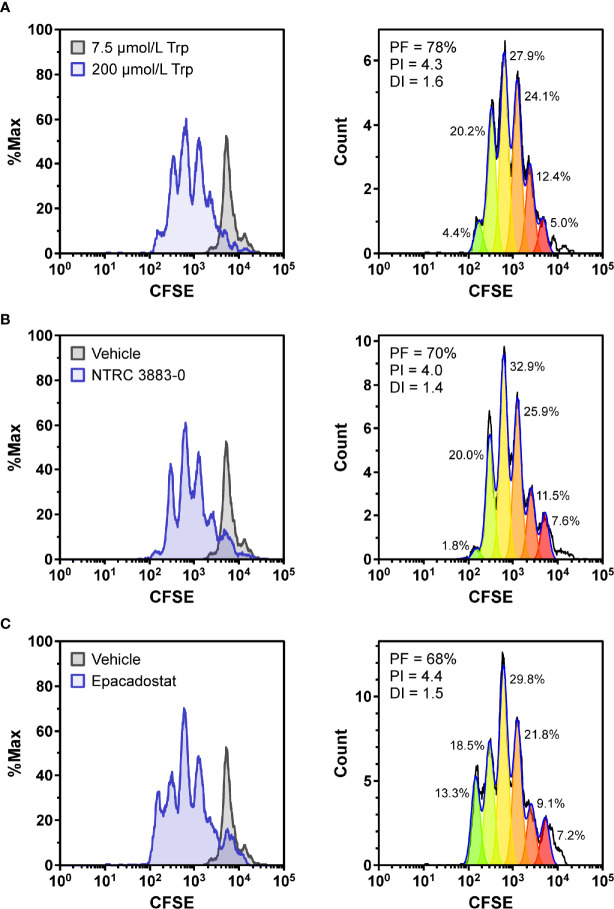
Co-culture assays of HEK-hIDO1 cells with lymphocytes from a healthy donor. Proliferation of CFSE-labeled CD8-positive T cells co-cultured for 5 days with HEK-hIDO1 cells upon addition of **(A)** 200 µM Trp as a positive control for T cell proliferation, **(B)** 10 µM NTRC 3883-0, and **(C)** 1 µM epacadostat. Each panel shows an overlay with CD8-positive T cells co-cultured for 5 days with HEK-hIDO1 cells upon addition of 7.5 µM Trp and vehicle (left), and the cell proliferation analysis (right). PF, precursor frequency (the percentage of cells from the original population that have proliferated); PI, proliferation index (the average number of cell divisions undergone by the proliferating cell population); DI, division index (the average number of cell divisions undergone by the entire cell population). The percentages indicate the percentage of the total amount of cells in each generation.

To determine whether NTRC 3883-0 can modulate immune cell activity *in vitro*, co-culture experiments of HEK-hIDO1 cells with peripheral blood mononuclear cells (PBMCs) from healthy donors were performed (**Figure 2**). Since T cell anergy is reported to occur only at Trp concentrations of 1 µM and lower (6, 24), the cells were co-cultured in Trp-free medium supplemented with a low concentration of Trp (*i.e.*, 7.5 µM Trp). Cytotoxic T cell proliferation was determined by labeling PBMCs with carboxyfluorescein succinimidyl ester (CFSE), a fluorescent dye that dilutes upon proliferation of cells, followed by analysis of CD8-positive T cells by flow cytometry. CD8-positive T cells stopped to proliferate when co-cultured with HEK-hIDO1 cells in medium containing 7.5 µM Trp (**Figure 2A**). When a high concentration of Trp (200 µM) was added to the co-culture with HEK-hIDO1 cells, the CD8-positive T cells continued to proliferate (**Figure 2A**). This indicates that the inhibitory effect of IDO1 is caused by depletion of Trp and is not due to the production of an immunosuppressive metabolite. Addition of 10 µM NTRC 3883-0 to co-cultures of HEK-hIDO1 and PBMCs at low Trp concentration relieved the inhibitory effect of IDO1 expression on T cell proliferation (**Figure 2B**), analogous to the effect of treatment with 1 µM epacadostat (**Figure 2C**). This demonstrates that NTRC 3883-0 can modulate immune cell function.

### *In Vivo* Target Modulation in Syngeneic Mouse Models

Next, we aimed to assess the *in vivo* activity of NTRC 3883-0 in a syngeneic mouse model. This first required the selection of a suitable IDO1-expressing, implantable cell line. There exist only a small number of mouse cancer cell lines as compared to human cancer cell lines, and only a few have been reported to express IDO1 *in vitro* (25, 26). In the colon carcinoma CT26 and melanoma B16F10 cell lines, the expression of IDO1 is low (25) or not detectable in cell culture (27). However, the expression of *IDO1* in CT26 cells is strongly increased after implantation in syngeneic mice (25), while implanted B16F10 cells have been found to promote IDO1 expression in dendritic cells (28). This indicates that *IDO1* gene expression can be induced by the tumor microenvironment, which is substantiated by studies with mice deficient for IFNγ or depleted of CD8-positive T cells indicating that upregulation of IDO1 in the tumor microenvironment is regulated by IFNγ released by CD8-positive T cells (29).

We initially selected the CT26 model to assess the *in vivo* efficacy of NTRC 3883-0, since this model has previously been used by others to evaluate several IDO1 inhibitors, including epacadostat (25). The effect of treatment with NTRC 3748-0 (**Table 1**), NTRC 3883-0 and epacadostat was evaluated in two CT26 mouse model experiments performed at different contract research organizations (**Figure S3**). While the first study reported inhibition of tumor growth with both NTRC 3748-0 and epacadostat (**Figure S3A**), no inhibition of tumor growth was found in the second study with either NTRC 3883-0 or epacadostat (**Figure S3B**). The tumors from both studies were harvested and analyzed for *mIDO1* gene expression by qPCR and Kyn levels by liquid chromatography-tandem mass spectrometry (LC-MS/MS). In both studies, *mIDO1* gene expression was detected in most, but not all CT26-derived tumors, while the expression levels were low and varied considerably among the mice (**Figures S3C, D**). Nonetheless, in both studies a similar, considerable reduction of Kyn levels was found in the tumor (**Figures S3E, F**), indicating target modulation regardless of tumor growth inhibition.

**Figure 3:**
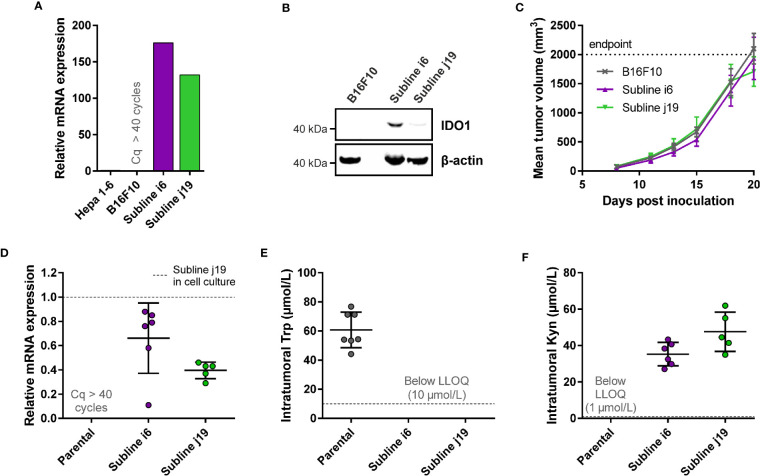
Development of a syngeneic mouse model using a B16F10 melanoma cell line stably overexpressing *mIDO1*. **(A)** Analysis of *mIDO1* mRNA levels by qPCR in the parental B16F10 cell line and two *mIDO1*-transfected stable sublines (i6 and j19). mRNA levels were normalized for the expression of three housekeeping genes (*ACTB*, *GAPDH*, and *RPL37*), and scaled based on the hepatoma Hepa 1-6 cell line stimulated with IFNγ (included as a positive control). **(B)** Immunoblot analysis of the parental B16F10 cell line and *mIDO1*-overexpressing sublines. **(C)** Mean tumor volume after implantation of the B16F10-mIDO1 i6 and j19 sublines in B6D2F1 mice. Results are expressed as mean ± SEM. **(D)** Analysis of *mIDO1* mRNA levels in the tumor tissues by qPCR. mRNA levels are scaled based on the mRNA level of the B16F10-mIDO1 subline j19 in cell culture. **(E)** Intratumoral Trp and **(F)** Kyn levels as determined by LC-MS/MS. LLOQ in **(E, F)** indicates the lower limit of quantification of the experiment. Results of **(D–F)** are expressed as mean ± SD.

With the aim to establish a mouse model with stable *mIDO1* gene expression, we next evaluated the use of an *mIDO1*-overexpressing cell line for the generation of a mouse model. We therefore generated stable sublines of the B16F10 melanoma cell line overexpressing mouse IDO1 (B16F10-mIDO1) (**Figure 3**). Transfection of full-length *mIDO1* cDNA followed by subcloning resulted in two stable sublines with different mIDO1 mRNA and protein levels (**Figures 3A, B**). The Trp-catabolizing activity of the sublines could be inhibited with NTRC 3883-0 and epacadostat (**Table 2**), while the selective TDO inhibitor NTRC 3531-0 was inactive (*i.e.*, IC_50_ > 31.6 µM). Whereas NTRC 3883-0 and epacadostat inhibited mIDO1 activity in a biochemical assay with similar potency (**Table 2**), NTRC 3883-0 was 5 times more potent compared to epacadostat in the cell-based B16F10-mIDO1 assay (**Table 2**).

To determine the effect of *mIDO1* expression on tumor growth, the parental B16F10 cell line and the two stable *mIDO1*-overexpressing sublines were implanted into female B6D2F1 syngeneic mice. The tumors grew rapidly, but there was no difference in growth rate between tumors derived from the parental cell line and the *mIDO1*-overexpressing sublines (**Figure 3C**). After sacrifice of the mice, the tumors were collected and analyzed for *mIDO1* gene expression by qPCR, while the Trp and Kyn levels were measured by LC-MS/MS. *mIDO1* expression was maintained in all tumors collected from the B16F10-mIDO1 sublines i6 and j19, with the mRNA levels in the tumors deviating on average only 2.0- and 2.5-fold from their respective cell lines in culture (**Figures 3A, D**). *mIDO1* expression resulted in strongly reduced Trp and increased Kyn levels (**Figures 3E, F**), indicating that the model could be suitable for pharmacodynamic studies of IDO1 inhibitors. The subline j19 was chosen for further experiments, as this subline showed the least variation in mRNA levels (**Figure 3D**) and induced on average the highest increase in kynurenine levels in the tumors (**Figure 3F**).

**Figure 4:**
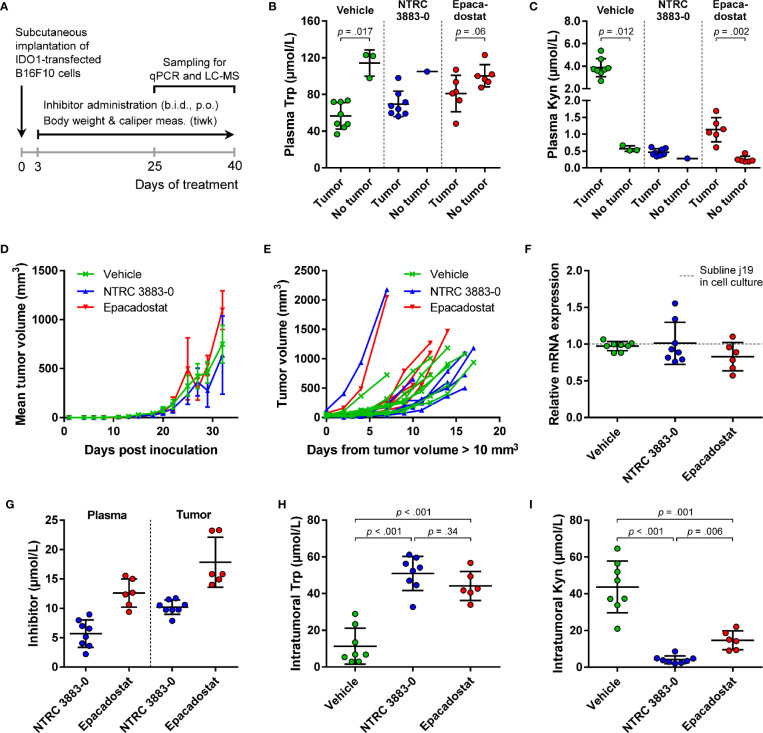
*In vivo* effect of NTRC 3883-0 and epacadostat in the B16F10-mIDO1 mouse model. **(A)** Experimental schedule of the intervention study with prophylactic administration of IDO1 inhibitors. **(B)** Analysis of Trp and **(C)** Kyn levels in plasma by LC-MS/MS. Results of the Mann-Whitney U test are indicated in the panels. Mice treated with NTRC 3883-0 were excluded from the statistical analysis as this group contained only one mouse without a tumor. Trp and Kyn levels are expressed as mean ± SD. **(D)** Mean tumor volume and **(E)** individual tumor volume of mice treated with vehicle, NTRC 3883-0, or epacadostat. The time scale in **(E)** takes into account the variation in time until tumors were established (defined as a tumor volume > 10 mm^3^). **(F)** Analysis of *mIDO1* mRNA levels in the tumor tissues by qPCR. mRNA levels were normalized for the expression of three housekeeping genes (*ACTB*, *GAPDH*, and *RPL37*), and scaled based on the mRNA level of the B16F10-mIDO1 subline j19 in cell culture. **(G)** Analysis of plasma and intratumoral inhibitor levels by LC-MS/MS. **(H)** Analysis of intratumoral Trp and **(I)** Kyn levels by LC-MS/MS. Welch’s ANOVA showed an overall effect of IDO1 inhibitor treatment on both Trp and Kyn levels (*p* >.001 for both inhibitors). Games-Howell *post hoc* results are indicated in the panels. Results in **(F–I)** are expressed as mean ± SD.

For evaluation of the *in vivo* effect of NTRC 3883-0 in the B16F10-mIDO1 mouse model, NTRC 3883-0 and epacadostat were both administered twice daily at 100 mg/kg. Dosing of the IDO1 inhibitors was started prior to establishment of the tumor (*i.e.*, prophylactic administration) (**Figure 4A**), based on previous experience of Charles River Laboratories, where the study was performed. In 22 out of the 32 animals, a tumor developed from the grafted B16F10-mIDO1 cells. Comparison of plasma Trp and Kyn levels among the mice revealed that vehicle-treated, tumor-bearing animals had significantly lower plasma Trp and higher plasma Kyn levels compared to non-tumor bearing animals (**Figures 4B, C**). This indicates that there is a direct effect of *mIDO1* expression in the tumors on systemic Trp and Kyn concentrations. The effect was diminished upon IDO1 inhibitor treatment (**Figures 4B, C**), indicating effective IDO1 inhibition. Treatment with NTRC 3883-0 or epacadostat had no effect on tumor growth in comparison to vehicle-treated animals (**Figure 4D**), although a delay in tumor growth could be observed when correcting the tumor growth data for the variation in time until tumor establishment (**Figure 4E**). After sacrifice of the mice, qPCR analysis revealed that the *mIDO1* expression in the tumors was remarkably stable (**Figure 4F**). Comparison of inhibitor concentrations revealed that NTRC 3883-0 reached on average 1.8 and 2.2 times lower levels in plasma and the tumors, respectively, compared to epacadostat (**Figure 4G**). IDO1 inhibitor treatment resulted in significantly increased intratumoral Trp levels and reduced Kyn levels (**Figures 4H, I**). Treatment with NTRC 3883-0 resulted in 3.6 times lower levels of Kyn compared to epacadostat, while it was also slightly more efficient in increasing Trp (**Figures 4H, I**). We conclude that NTRC 3883-0 can modulate IDO1 activity *in vivo*, thus showing target engagement.

### Modulation of IDO1 Activity in Primary Ovarian Cancer Cells

We next extended our studies to primary, ascites-derived cells of ovarian cancer patients. In ovarian cancer, IDO1 has been related to disease progression (30), chemotherapy resistance (9) and impaired survival (9, 31), based on expression analysis of IDO1 by *in situ* hybridization and immunohistochemistry of tumor specimens (9, 30, 31). Patients with advanced ovarian cancer often present with high volumes of malignant ascites, which is routinely collected for diagnostic purposes or relieve of symptoms. Ascites is a potential source for biomarkers to monitor disease progression and to determine chemotherapy response *ex vivo*.

**Figure 5:**
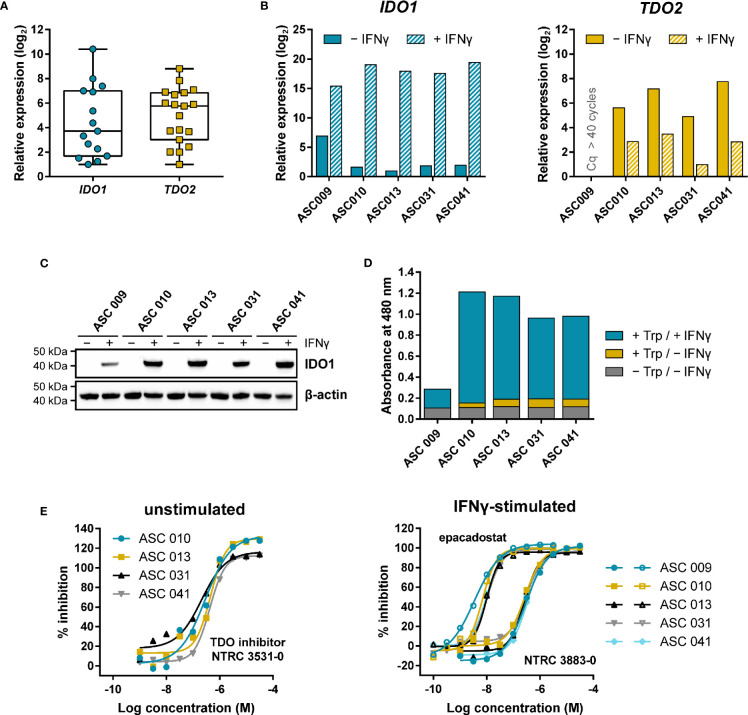
Expression and modulation of IDO1 and TDO in adherent cell samples isolated from the ascites of ovarian cancer patients. **(A)** Tukey boxplot of basal *IDO1* and *TDO2* gene expression as determined by qPCR in adherent cell samples cultured for two to three passages. For *IDO1*, only the fifteen samples with detectable expression are shown. mRNA levels were normalized for the expression of the *ACTB* housekeeping gene, and scaled based on the lowest expressing samples. **(B)** Analysis of *IDO1* and *TDO2* mRNA levels in five adherent cell samples in the absence and presence of IFNγ. mRNA levels in **(B)** were normalized for the expression of the *ACTB* and *RPS18* housekeeping genes, and scaled based on the lowest expressing samples. **(C)** IDO1 protein levels measured in the same sample subset as in **(B)**. TDO protein levels of the samples could not be analyzed due to the lack of a suitable antibody. **(D)** Trp-catabolizing activity in the same sample subset as in **(B)**. The activities are displayed as the absorbance measured using the pDMAB assay, and are based on three replicate experiments for ASC 009, ASC 031, and ASC 041, and two replicate experiments for ASC 010 and ASC 013. **(E)** Inhibition of the Trp-catabolizing activity in unstimulated samples by the selective TDO inhibitor NTRC 3531-0, and in IFNγ-stimulated samples by NTRC 3883-0 (closed symbols) and epacadostat (open symbols). The corresponding IC_50_ values are listed in **Table S5**.

Adherent cell samples were isolated from the ascites collected from nineteen ovarian cancer patients with different histologies (**Table S3**). The presence of tumor cells in these samples was confirmed by qPCR analysis of the ovarian cancer marker genes *MUC16* and *HE4* (**Figure S4**), while five of the nineteen cell samples were additionally analyzed for cell surface expression of mucin-16 and epithelial cell adhesion molecule (EpCAM) by flow cytometry (**Figure S5**; **Table S4**). Basal *IDO1* gene expression could be detected by qPCR in fifteen of the nineteen samples, while basal *TDO2* expression was found in all nineteen samples (**Figure 5A**).

Five of the nineteen samples were further analyzed for *IDO1* and *TDO2* gene expression, IDO1 protein levels, and Trp-catabolizing activity both in the presence and absence of IFNγ, a known inducer of *IDO1* gene expression (**Figures 5B–D**). qPCR analysis revealed that four out of the five samples showed comparable, relatively low basal *IDO1* mRNA levels, which were highly increased (*i.e.*, on average 120,000-fold) upon stimulation with IFNγ (**Figure 5B**). In contrast, 40-fold higher basal *IDO1* expression was found in ASC 009, which was increased by 360-fold upon IFNγ stimulation (**Figure 5B**). Immunoblot analysis of IDO1 revealed a consistent pattern (**Figure 5C**). Analysis of the *TDO2* mRNA levels showed clear basal *TDO2* expression in four samples, albeit with slightly more variation among the samples compared to *IDO1*, while no *TDO2* expression was detected in ASC 009 either with or without stimulation (**Figure 5B**). Notably, *TDO2* expression was found to be consistently downregulated 7- to 30-fold upon stimulation with IFNγ (**Figure 5B**).

Assessment of the Trp-catabolizing activity in the samples showed basal activity in four out of the five samples (**Figure 5D**; + Trp/− IFNγ). Treatment with the TDO-selective inhibitor NTRC 3531-0 resulted in a concentration-dependent inhibition of this activity with an average IC_50_ of 300 nM (**Figure 5E**; **Table S5**). In contrast, NTRC 3883-0 and epacadostat showed no or only minimal inhibition (**Table S5**). This indicates that the Trp-catabolizing activity in the absence of stimulation can be solely attributed to the activity of TDO, and not IDO1, despite the constitutive expression of *IDO1* observed in most samples (**Figures 5A, B**). Stimulation of the ascites cell samples with IFNγ induced a further increase in Trp-catabolizing activity (**Figure 5D**; + Trp/+ IFNγ), with a consistent pattern as that observed for IDO1 mRNA and protein levels. In the stimulated samples, treatment with either NTRC 3883-0 or epacadostat resulted in potent inhibition of the Trp-catabolizing activity with IC_50_ values of respectively 261 nM and 8.3 nM (**Figure 5E**; **Table S5**), which are in close agreement with the potencies found in the A375- and HEK-hIDO1-based assays (**Table 2**). Moreover, the Trp-catabolizing activity could not be inhibited by the TDO inhibitor NTRC 3531-0 (*i.e.*, IC_50_ > 31.6 μM). This demonstrates that the IFNγ-dependent Trp-catabolizing activity of the primary patient-derived cancer cells can be attributed to IDO1 activity, and can be selectively inhibited by NTRC 3883-0.

## Discussion

IDO1 is a key regulator of the immune modulatory activity of Trp and is therefore a candidate drug target to increase the efficacy of checkpoint inhibitor therapy. We have described the pharmacological characterization of a novel, selective IDO1 inhibitor, NTRC 3883-0, which was profiled in various biochemical and cell-based assays alongside epacadostat. In a co-culture assay with healthy donor lymphocytes, our data confirm that IDO1 expression suppresses cytotoxic T cell proliferation *in vitro* by decreasing the Trp concentration. Treatment with an IDO1 inhibitor (*i.e.*, NTRC 3883-0 or epacadostat) abolished this inhibitory effect.

In search of a mouse model to additionally profile the *in vivo* efficacy of NTRC 3883-0, we initially chose the frequently used CT26 mouse model, which is described to endogenously express *IDO1* when grafted in mice (25). However, this model was found to be unreliable based on unreproducible tumor growth inhibition results among our two similar studies. Moreover, strong variation in the intratumoral *IDO1* mRNA levels was found, with some mice having undetectably low expression. To abolish the potential effect of the low and strongly variable *IDO1* expression on the reliability of the efficacy model, we developed a syngeneic mouse model using B16F10 melanoma cells stably overexpressing *mIDO1*. While we have demonstrated strong target modulation in this model upon treatment with NTRC 3883-0, we did not observe an effect of IDO1 expression or IDO1 inhibitor treatment on the tumor growth rate. This is in contrast with the results obtained by Holmgaard and coworkers, who describe the first use of this model, and who describe an increased tumor growth rate after IDO1 overexpression, which is reduced by IDO1 inhibitor treatment (27). The reason for this discrepancy is unclear, but may be related to subtle differences in the *mIDO1*-overexpressing or parental cell lines, the mouse strains used or the breeding conditions. Nonetheless, based on the demonstrated target modulation observed for both NTRC 3883-0 and epacadostat, the B16F10-mIDO1 model has proven suitable for application in pharmacodynamic studies of IDO1 inhibitors.

When our studies were still in progress, negative clinical results were reported on several IDO1 inhibitors, including epacadostat (17), EOS200271/PF-0684003 (32), and navoximod (NLG-919) (33). Several experts have commented on these results, in particular on those obtained in the phase III combination trial of epacadostat with pembrolizumab (34–36). Among the potential causes mentioned for the negative results, the dosing strategy was prominently discussed, since the dose of epacadostat may have been too low to obtain sufficient target coverage in the ECHO-301/KEYNOTE-252 clinical trial. The lack of a good patient stratification strategy was additionally criticized, since patients included in this study had not been selected on the basis of IDO1 expression, although tumor *IDO1* gene expression was assessed as part of the study (17). A third possibility raised is that TDO may compensate for inhibition of IDO1, and it has been suggested that dual IDO1/TDO inhibitors may result in more effective anti-tumor immunity (37).

Learning from these lessons, NTRC 3883-0 was not pursued for further clinical development. This was decided based on allometric scaling indicating that NTRC 3883-0 is unlikely to reach sufficient target coverage of human IDO1 at acceptable dose levels in cancer patients (**Figure S2**; **Table S2**). However, because of its favorable inhibitory potency on mouse IDO1, its better selectivity in biochemical assays, and its higher degree of *in vivo* target modulation compared to epacadostat, NTRC 3883-0 may become an important tool compound to study the role of IDO1 in mouse disease models. In addition to cancer models, this may support research on neurodegenerative and infectious diseases, for which a role of IDO1 has also been implicated (38, 39).

In the phase III clinical trial of epacadostat, *IDO1* gene expression in tumor samples was quantified by *in situ* hybridization (17). Our studies with primary ovarian cancer cell cultures isolated from ascites indicate that determination of basal *IDO1* gene expression may not be sufficient. We found that only after stimulation with IFNγ, modulation of Trp-catabolizing activity by IDO1 inhibitors could be observed. We propose that functional cell-based assays for IDO1 are included as diagnostic tools to select patients most likely to respond to IDO1 inhibitor therapy. Our research demonstrates for the first time the use of malignant ascites as a source of biomarkers for clinical research on IDO1. The use of cells from ascites has a major advantage over the use of surgical biopsies or organoid cultures (40). The collection of ascites is minimally invasive and assays to determine the activity of compounds can be performed *ex vivo* prior to the start of therapy.

Notably, we could detect *TDO* gene expression in all nineteen primary ovarian cancer cell cultures examined. By making use of a selective inhibitor, we demonstrated basal TDO activity in four cultures. Thus far, only a few cancer cell lines that express TDO constitutively are known, while many cell lines express IDO1 upon stimulation with IFNγ (8, 41). TDO has been implicated in cancer, but its role is not well understood. Based on its high *K*_M,Trp_ of 190 µM, it is unlikely that TDO contributes to the regulation of the low Trp concentrations that induce T cell anergy, which are around 1 µM and lower (6, 24). However, TDO may regulate immune function through the metabolite Kyn, which is a ligand of the aryl hydrocarbon receptor (42). Furthermore, roles of TDO in tumor metastasis (43) and angiogenesis (44) have been described. Because of the different roles of IDO1 and TDO in cancer, it is unlikely that TDO expression may have compensated for IDO1 inhibition in the ECHO-301/KEYNOTE-252 trial. Therefore, there is no strong rationale for the development of dual IDO1/TDO inhibitors, since the possible beneficial effects of dual inhibition have to be balanced with the risk of interfering with the role of TDO in Trp homeostasis.

While many companies have de-prioritized their IDO1 programs, the development of the selective and potent IDO1 inhibitor linrodostat has continued. Linrodostat has demonstrated safety and good target coverage upon once-daily oral dosing in a phase I/IIa study (45), and is currently investigated in a Phase III combination trial with the anti-PD1 immunotherapeutic nivolumab and chemotherapy for advanced bladder cancer (46). Preliminary clinical response data hold promise for IDO1 as a valid drug target for immunotherapy (46). A positive outcome will undoubtedly fuel interest in the development of additional IDO1 inhibitors with differentiating properties. The novel IDO1 inhibitor scaffold described here as well as our *in vitro* and *in vivo* pharmacological models may support these R&D activities.

## Data Availability Statement

The original contributions presented in the study are included in the article/**Supplementary Material**. Further inquiries can be directed to the corresponding author.

## Ethics Statement

The animal study was reviewed and approved by Ethics Committee for Animal Experimentation at Charles River Laboratories Morrisville, NC, and Ethics Committee for Animal Experimentation at ProQinase Freiburg, Germany.

## Author Contributions

Conceptualization: YG, JM, JU, RB, GZ. Resources: RB, GZ. Funding acquisition: RB, GZ. Investigation: YG, JM, AD, MM, NW-S, DV-P, WM, MP, JW, JS, FC, JO, AA, LM, JU, RB, GZ. Visualization: YG. Methodology: AD, DV-P, WM. Writing – original draft: YG, GZ. Writing—review and editing: JU. All authors contributed to the article and approved the submitted version.

## Funding

The IDO1 screen was performed in the context of the European Lead Factory, a project which has received support from the Innovative Medicines Initiative Joint Undertaking under grant agreement n’ 115489, resources of which are composed of financial contribution from the European Union’s Seventh Framework Programme (FP7/2007-2013) and EFPIA companies’ in kind contribution.

## Conflict of Interest

RB and GZ are managing directors and shareholders of Netherlands Translational Research Center B.V. YG, JM, AD, MM, NW-S, DV-P, WM, MP, JW, JS, FC and JU were employed by Netherlands Translational Research Center B.V.

The remaining authors declare that the research was conducted in the absence of any commercial or financial relationships that could be construed as a potential conflict of interest.

## Abbreviations

b.i.d., twice daily; CFSE, carboxyfluorescein succinimidyl ester; CYP, cytochrome P450; ECL, enhanced chemiluminescence; EpCAM, epithelial cell adhesion molecule; hWB, human whole blood; IC_50_, half-maximal inhibitory concentration; IDO1, indoleamine 2,3-dioxygenase 1; Kyn, kynurenine; LC-MS/MS, liquid chromatography-tandem mass spectrometry; PBMCs, peripheral blood mononuclear cells; pDMAB, 4-(dimethylamino)benzaldehyde; p.o., oral; P/S, penicillin/streptomycin; q.d., once daily; TDO, tryptophan 2,3-dioxygenase; Trp, tryptophan.
