# Supplementary material for: Targeting Indoleamine 2,3-Dioxygenase in Cancer Models Using the Novel Small Molecule Inhibitor NTRC 3883-0
Source: Front Immunol. 2021 Jan 28;11:609490. doi: 10.3389/fimmu.2020.609490 (PMC7876453; doi:10.3389/fimmu.2020.609490)
Supplement: Supplementary file 1 [file DataSheet_1.docx]

Supplementary Material

**Supplementary Tables**

**Table S1.** DNA sequences of oligonucleotide primers used for quantitative real-time PCR analysis of *IDO1,* *TDO2* and ovarian cancer gene markers in cell lines, patient samples and tumors.

| **Gene** | **Species** | **Direction** | **Sequence** |
| --- | --- | --- | --- |
| *IDO1^a^* | human | forward | GGCTTTGCTCTGCCAAATCC |
|  | human | reverse | TTCTCAACTCTTTCTCGAAGCTC |
| *IDO1^b^* | human | forward | TTGCTAAAGGCGCTGTTGGA |
|  | human | reverse | GTCTGATAGCTGGGGGTTGC |
| *TDO2* | human | forward | CTTAGTAAAGGTGAAAGACGG |
|  | human | reverse | GTCCATAAGAGAAGTCAGCA |
| *MUC16* | human | forward | CACAGACAACGTCATGCAGC |
|  | human | reverse | TGGGAGTTGTAGGAGGCTCA |
| *HE4* | human | forward | CAAGAGTGCGTCTCGGACAG |
|  | human | reverse | TTAATGTTCACCTGGGGGCA |
| *ACTB* | human | forward | CAAGAGATGGCCACGGCTGCTTCCA |
|  | human | reverse | GCATGGAGTTGAAGGTAGTTTCG |
| *RPS18* | human | forward | GACAACAAGCTCCGTGAAGA |
|  | human | reverse | AGAAGTGACGCAGCCCTCTA |
| *IDO1* | mouse | forward | GGCTTTGCTCTACCACATCCAC |
|  | mouse | reverse | TAGCCACAAGGACCCAGGG |
| *ACTB* | mouse | forward | GGCTGTATTCCCCTCCATCG |
|  | mouse | reverse | CCAGTTGGTAACAATGCCATGT |
| *GAPDH* | mouse | forward | ACGGATTTGGTCGTATTGGG |
|  | mouse | reverse | CGCTCCTGGAAGATGGTGAT |
| *RPL37* | mouse | forward | TCTGTGGCAAGACCAAGATG |
|  | mouse | reverse | GACAGCAGGGCTTCTACTGG |

*^a^* Primer set used in Figure 5A; *^b^* Primer set used in Figure 5B

**Table S2.** Pharmacokinetic parameters of NTRC 3883-0 observed in mouse, rat, monkey and dog, and predicted for humans using allometric scaling. Data in bold are measured, while data in italics are calculated or predicted (for humans). The clearance (Cl) and volume of distribution at steady state (V_ss_) in humans are predicted based on extrapolation of the parameters in the four species as shown in Supplementary Figure S2. No correction was made for plasma protein binding, as this data was not available for monkey. The half-life (t_1/2_) in humans was predicted as V_ss_/Cl. Based on the predicted parameters in humans and assuming a 100% bioavailability, a minimum dose of 1725 mg per day would be required to reach a target concentration of 1 μM (IC_90_ in dog whole blood assay) on average during the dosing interval. However, considering the high expected clearance in humans, it is likely that the inhibitor undergoes considerable first-pass metabolism and therefore even higher doses would be required to reach the target level. ND = not determined.

| **Species** | **Mouse** | **Rat** | **Monkey** | **Dog** | ***Human (predicted)*** |
| --- | --- | --- | --- | --- | --- |
| **Body weight (kg)** | **0.036** | **0.29** | **3.0** | **10** | *70* |
| **Cl (mL/h/kg)** | **1110** | **1143** | **2222** | **1717** |  |
| *Cl (mL/h)* | *40* | *331* | *6666* | *17170* | *171137* |
| **V_ss_ (mL/kg)** | **3627** | **1428** | **2246** | **1362** |  |
| *V_ss_ (L)* | *0.131* | *0.417* | *6.738* | *13.62* | *79.8* |
| **t_1/2_ (h)** | **5.2** | **3.3** | **2.4** | **2.8** | *0.47* |
| **Bioavailability (F)** | **ND** | **91%** | **49%** | **32%** |  |
| **Plasma protein binding (%)** | **54** | **85** | **ND** | **79** | **74** |

**Table S3.** Tumor histopathology of ovarian cancer patients from whom ascites cell cultures were derived. The disease stage is based on the International Federation of Gynecology and Obstetrics (FIGO) staging system. Low grade and high grade serous ovarian cancer are indicated as LGSOC and HGSOC.

| **Sample** | **FIGO stage** | **Histopathology** |
| --- | --- | --- |
| ASC 005 | IIIc | HGSOC |
| ASC 006 | IIIc | mucinous |
| ASC 009 | IIIc | LGSOC |
| ASC 010 | IIIc | HGSOC |
| ASC 011 | IIIc | serous |
| ASC 012 | IV | HGSOC |
| ASC 013 | IIIc | HGSOC |
| ASC 014 | IIIc | HGSOC |
| ASC 016 | IVa | HGSOC |
| ASC 020 | IIIc | HGSOC |
| ASC 022 | IIIc | serous |
| ASC 024 | IVb | HGSOC |
| ASC 025 | IVb | HSCOC |
| ASC 027 | IVa | HGSOC |
| ASC 031 | IIIc | HGSOC |
| ASC 035 | IIIc | serous |
| ASC 036 | IIIc | HGSOC |
| ASC 041 | IVa | HGSOC |
| ASC 045 | IV | HGSOC |

**Table S4.** Quantification of the expression of mucin-16 and EpCAM on ovarian cancer cell samples by flow cytometry. Expression was quantified by determining the ratio of the median fluorescence intensity (MFI) of the peak corresponding to the surface marker and the peak corresponding to the isotype control antibody. Corresponding flow cytometry peak patterns are shown in Figure S5.

|  |  | **Doubling time (h)** | **MFI ratio** | |
| --- | --- | --- | --- | --- |
| **Sample** | **Passage** |  | **Mucin-16** | **EpCAM** |
| ASC 009 | P+11 | 70 | 1.3 | 71.7 |
| ASC 010 | P+5 | 58 | 1.2 | 0.9 |
| ASC 013 | P+5 | 56 | 2.2 | 1.2 |
| ASC 031 | P+7 | 75 | 1.6 | 2.7 |
| ASC 041 | P+7 | 65 | 2.1 | 2.0 |
| SK-OV-3 | P+2 |  | 2.1 | 43.8 |

**Table S5.** Potency (IC_50_) in nmol/L of IDO1 inhibitors NTRC 3883-0 and epacadostat, and TDO inhibitor NTRC 3531-0 in unstimulated and IFNγ-stimulated adherent cell samples isolated from ascites of ovarian cancer patients. n indicates the number of experimental replicates. 95% confidence intervals are given within brackets.

|  |  | **unstimulated** | | |  | **IFNγ-stimulated** | | |
| --- | --- | --- | --- | --- | --- | --- | --- | --- |
| **Sample** | **n** | **NTRC 3883-0** | **epacadostat** | **NTRC 3531-0** |  | **NTRC 3883-0** | **epacadostat** | **NTRC 3531-0** |
| ASC 009 | 3 | - | - | - |  | 236 (156 – 352) | 3.7 (2.3 – 5.9) | > 31,600 |
| ASC 010 | 3 | > 31,600 | 9,640 (6,130 – 15,200) | 282 (268 – 297) |  | 294 (193 – 450) | 9.3 (4.5 – 19) | > 31,600 |
| ASC 013 | 2 | > 31,600 | 9,940 | 328 |  | 237 | 9.0 | > 31,600 |
| ASC 031 | 3 | > 31,600 | 8,630 (4,200 – 17,700) | 256 (157 – 416) |  | 271 (237 – 311) | 7.2 (5.3 – 9.7) | > 31,600 |
| ASC 041 | 3 | > 31,600 | 4,310 (3,320 – 5,590) | 344 (278 – 425) |  | 321 (174 – 589) | 7.4 (3.9 – 14) | > 31,600 |
| Mean |  | > 31,600 | 7,730 (5,250 – 11,400) | 300 (263 – 343) |  | 261 (232 – 293) | 8.3 (5.4 – 13) | > 31,600 |

**Supplementary Figures**


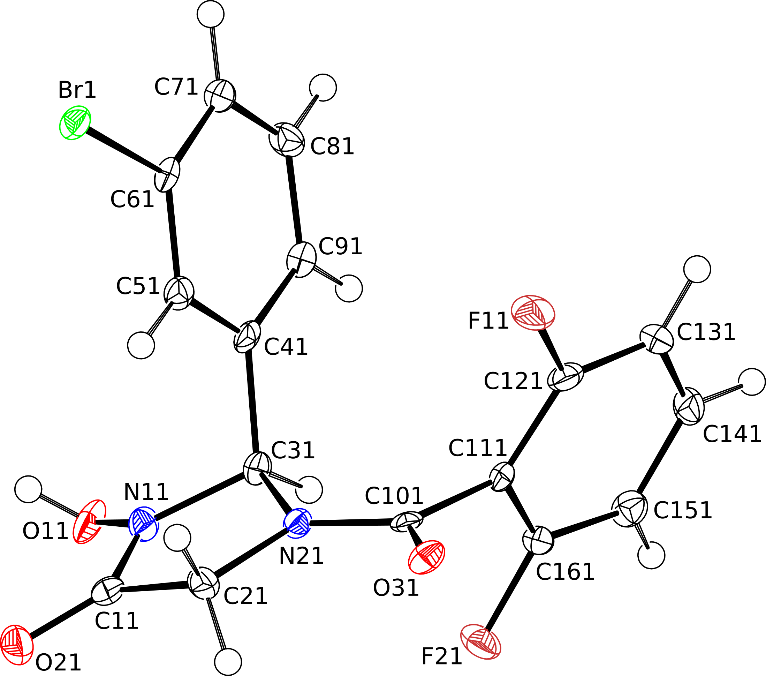


**Figure S1.** Crystal structure (ORTEP diagram) of NTRC 3883-0.


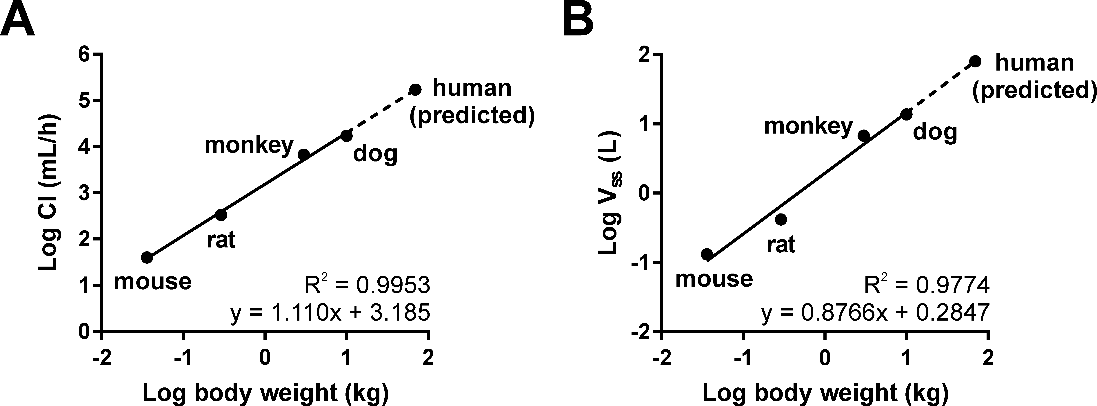


**Figure S2.** Allometric graphical plots for (**A**) clearance (Cl) and (**B**) volume of distribution at steady state (V_ss_) of NTRC 3883-0 in mouse, rat, monkey and dog and predicted parameters for human based on extrapolation. Values for the individual parameters are listed in Table S2.


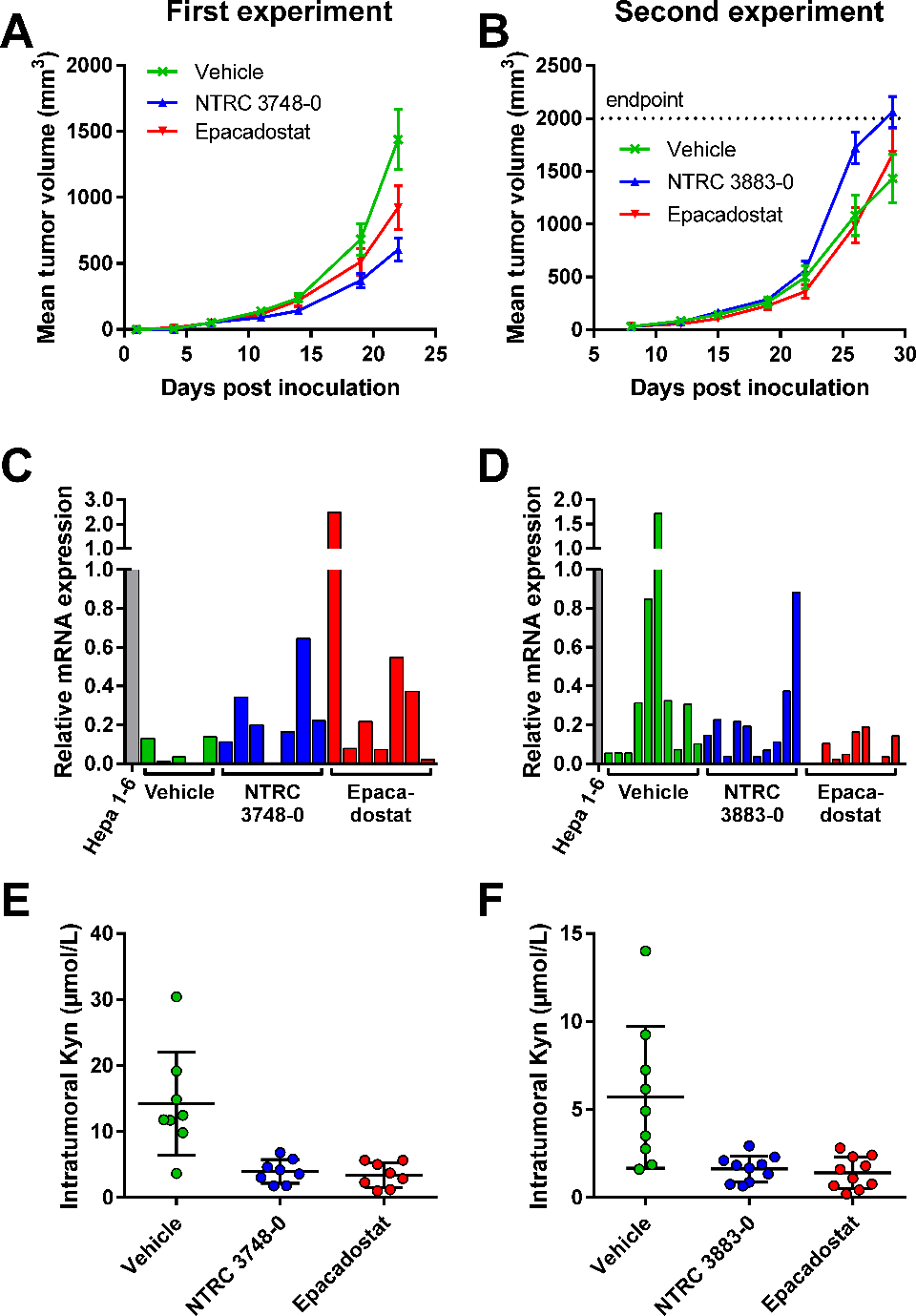


**Figure S3.** *In vivo* effect of NTRC 3748-0 (a racemic mixture of NTRC 3883-0 and its enantiomer), NTRC 3883-0 and epacadostat in two CT26 mouse model experiments. (**A**) Mean tumor volume of mice treated with vehicle, 50 mg/kg NTRC 3748-0 (b.i.d.) or 100 mg/kg epacadostat (q.d.) during the first study. (**B**) Mean tumor volume of mice treated with vehicle, 100 mg/kg NTRC 3883-0 (b.i.d.) or 100 mg/kg epacadostat (b.i.d.) during the second study. Results in panels A and B are expressed as mean ± SEM. (**C**) Analysis of *mIDO1* mRNA levels in the tumor tissues collected from the first study by qPCR. mRNA levels were normalized for the expression of two housekeeping genes (*ACTB* and *RPL37*)*,* and scaled based on the hepatoma Hepa 1-6 cell line stimulated with IFNγ (included as a positive control)*.* Five samples were excluded from the analysis due to poor cDNA quality. Missing bars represent mRNA levels below the detection limit (*i.e.*, Cq > 40 cycles). (**D**) Analysis of *mIDO1* mRNA levels in the tumor tissues collected from the second study, presented as for panel C. In both studies, the mRNA levels were low based on the average Cq values of 36 cycles for the first study (panel C) and 35 cycles for the second study (panel D). (**E**) Analysis of intratumoral Kyn levels by LC-MS/MS for the first study and (**F**) the second study. Results in panels E and F are expressed as mean ± SD.


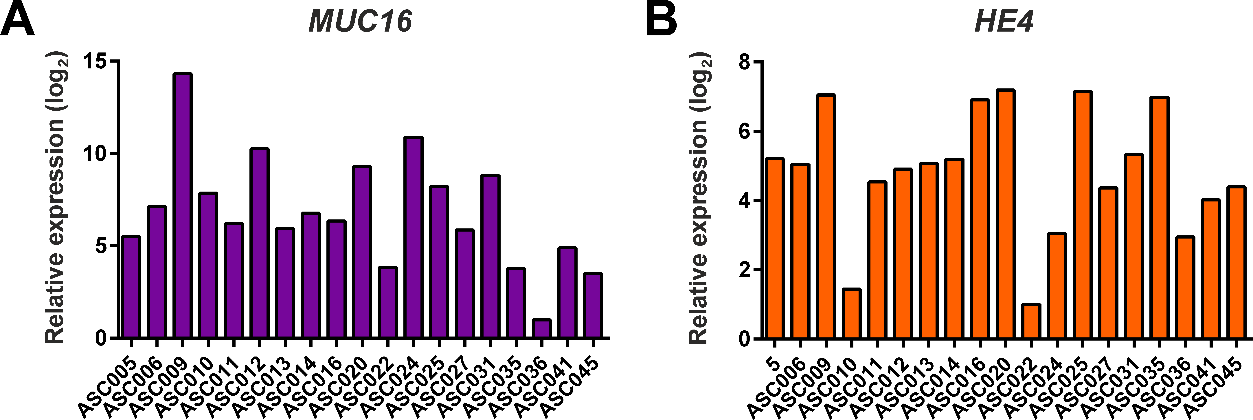


**Figure S4.** Expression analysis by qPCR of the ovarian cancer marker genes *MUC16* (**A**) and *HE4* (**B**) in cell cultures isolated from the malignant ascites of patients with advanced epithelial ovarian cancer. The same mRNA samples were used as in Figure 5A. mRNA levels were normalized for the expression of β-actin (*ACTB*) and ribosomal protein S18 (*RPS18*), and scaled based on the lowest expressing samples.


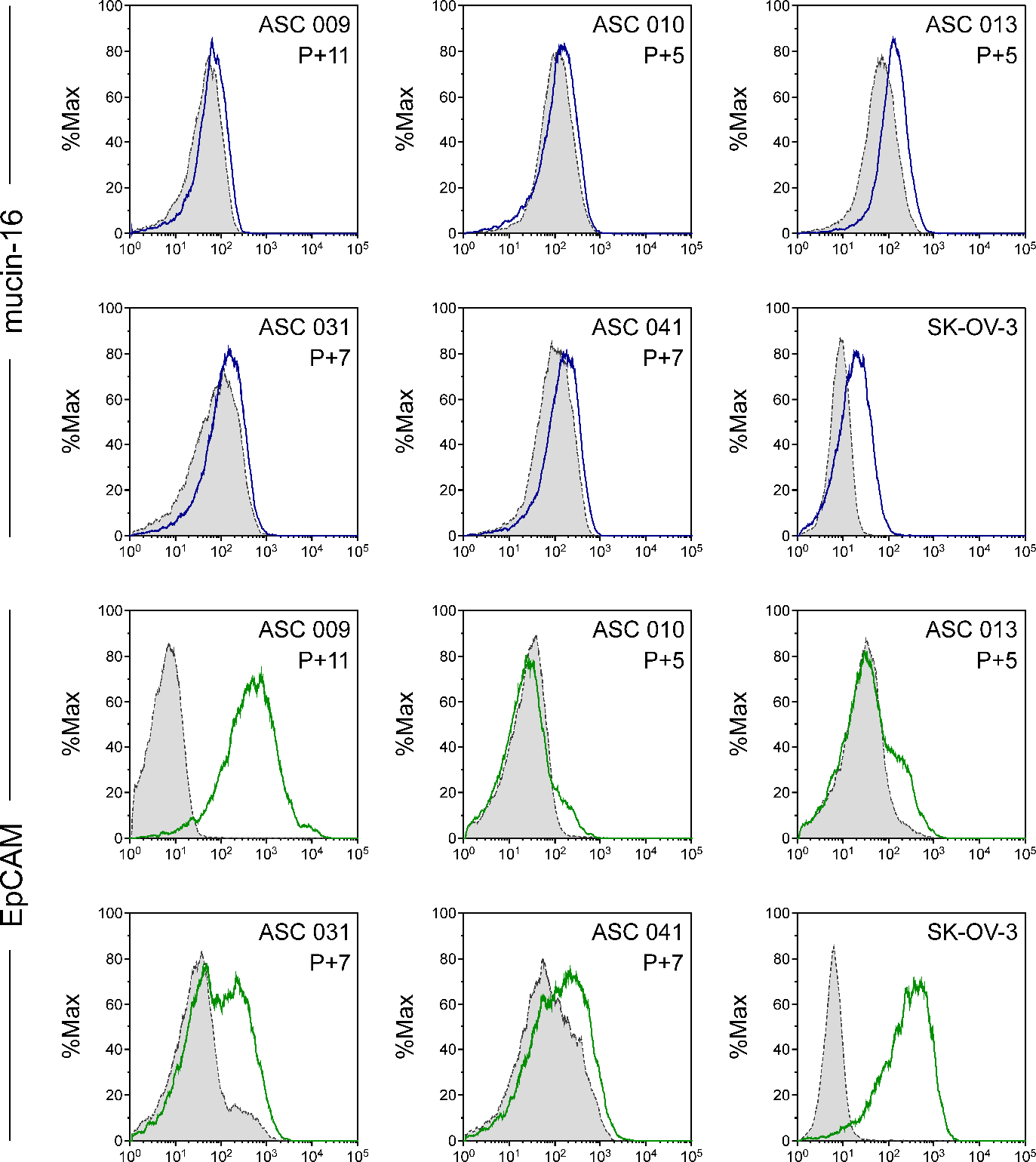


**Figure S5.** Analysis of the tumor cell markers mucin-16 and EpCAM on five patient-derived cell samples by flow cytometry. The passage number (P) at the time of analysis is indicated. Grey-shaded peaks represent the staining with isotype control antibodies. Expression was quantified by determining the ratio of the median fluorescence intensity (MFI) of the peak corresponding to the surface marker and the peak corresponding to the isotype control antibody (Table S4). The adenocarcinoma ovarian cancer cell line SK-OV-3 was included for reference.
